# Dual-Layer Spectral CT as Innovative Imaging Guidance in Lung Biopsies: Could Color-Coded Z-Effective Images Allow More Diagnostic Samplings and Biomarkers Information?

**DOI:** 10.3390/jcm12237426

**Published:** 2023-11-30

**Authors:** Filippo Piacentino, Federico Fontana, Giada Zorzetto, Angiola Saccomanno, Tonia Gatta, Chiara Recaldini, Francesca Franzi, Andrea Imperatori, Nicola Rotolo, Andrea Coppola, Manuela Minenna, Roberto Minici, Velio Ascenti, Gianluca Tripodi, Antonio Bottari, Domenico Laganà, Anna Maria Ierardi, Gianpaolo Carrafiello, Fausto Sessa, Giulio Carcano, Giorgio Ascenti, Massimo Venturini

**Affiliations:** 1 Department of Diagnostic and Interventional Radiology, Circolo Hospital and Macchi Foundation, Insubria University, 21100 Varese, Italy; federico.fontana@asst-settelaghi.it (F.F.); andrea.coppola@asst-settelaghi.it (A.C.); massimo.venturini@uninsubria.it (M.V.); 2 Department of Medicine and Technology Innovation (DiMIT), Insubria University, 21100 Varese, Italy; fausto.sessa@uninsubria.it (F.S.); giulio.carcano@asst-settelaghi.it (G.C.); 3 Department of Diagnostic and Interventional Radiology, Circolo Hospital, ASST Sette Laghi, 21100 Varese, Italy; giada.zorzetto@asst-settelaghi.it (G.Z.); chiara.recaldini@asst-settelaghi.it (C.R.); 4 Postgraduate School of Radiodiagnostics, Insubria University, 21100 Varese, Italy; asaccomanno@studenti.uninsubria.it (A.S.); tgatta@studenti.uninsubria.it (T.G.); 5 Division of Pathological Anatomy, Circolo Hospital and Macchi Foundation, Insubria University, 21100 Varese, Italy; francesca.franzi@asst-settelaghi.it; 6 Department of Medicine and Surgery, Circolo Hospital and Macchi Foundation, Insubria University, 21100 Varese, Italy; andrea.imperatori@asst-settelaghi.it (A.I.); nicola.rotolo@asst-settelaghi.it (N.R.); 7 Postgraduate School of Radiology Technician, Insubria University, 21100 Varese, Italy; manuela.minenna@studenti.uninsubria.it; 8 Radiology Unit, Department of Experimental and Clinical Medicine, University Hospital Mater Domini, Magna Graecia University of Catanzaro, 88100 Catanzaro, Italy; roberto.minici@unicz.it (R.M.); domenico.lagana@unicz.it (D.L.); 9 Postgraduate School of Diagnostic and Interventional Radiology, University of Milan, 20122 Milan, Italy; velio.ascenti@unimi.it; 10 Postgraduate School of Diagnostics and Interventional Radiology, University of Messina, 98122 Messina, Italy; gianluca.tripodi@unime.it; 11 Section of Radiological Sciences, Department of Biomedical Sciences and Morphological and Functional Imaging, University of Messina, Policlinico “G. Martino”, Via Consolare Valeria 1, 98122 Messina, Italy; antonio.bottari@unime.it (A.B.); giorgio.ascenti@unime.it (G.A.); 12 Interventional Radiology Unit, Department of Radiology, Foundation IRCCS Ca’ Granda-Ospedale Maggiore Policlinico, 20126 Milan, Italy; annamaria.ierardi@unimi.it (A.M.I.); gianpaolo.carrafiello@unimi.it (G.C.); 13 Department of General, Emergency and Transplants Surgery, Insubria University, ASST Dei Sette Laghi, 21100 Varese, Italy

**Keywords:** percutaneous biopsy, spectral CT, cone beam CT, Z-effective images, fusion imaging

## Abstract

The aim of the study was to try to obtain more information on diagnostic samplings and biomarkers using dual-layer spectral CT in lung biopsies. Lung biopsies were performed by merging images obtained with CBCT with those from spectral CT to use them as functional guidance, experimenting with double sampling to determine the difference between the area with a higher Z-effective number and that with a lower Z-effective number. Ten patients with large lung lesions on spectral CT were selected and underwent percutaneous transthoracic lung mass biopsy. Technical success was calculated. The percentage of neoplastic, inflammatory, fibrotic, necrotic cells, or non-neoplastic lung parenchyma was reported. The possibility of carrying out immunohistochemical or molecular biology investigations was analyzed. All lesions were results malignant in 10/10 samples in the Z_max_ areas; in the Z_min_ areas, malignant cells were found in 7/10 samples. Technical success was achieved in 100% of cases for Z_max_ sampling and in 70% for Z_min_ sampling (*p*-value: 0.2105). The biomolecular profile was detected in 9/10 (90%) cases in Z_max_ areas, while in 4/10 (40%) cases in Z_min_ areas (*p*-value: 0.0573). The advantage of Z-effective imaging would be to identify a region of the lesion that is highly vascularized and probably richer in neoplastic cells, thus decreasing the risk of obtaining a non-diagnostic biopsy sample.

## 1. Introduction 

Lung cancer is the most widespread neoplasm in men and the second in women [1].

Mortality is very high notwithstanding the many therapeutic approaches such as surgery [2,3,4], radiotherapy [5,6], ablation [7,8,9], chemotherapy [10,11], and immunotherapy [12,13], alone or combined according to the abscopal effect [14,15]. Screening programs have been largely developed in order to early diagnose small lung nodules and prevent distant metastases [16].

The use of imaging techniques, such as CT and PET/CT, has significantly improved the detection rate of pulmonary nodules [17]. 

Furthermore, in the era of personalized oncological therapies, the role of percutaneous biopsy is evolving, as the status of biomarkers allows for guiding therapeutic choices in many solid tumors, not only at the time of initial diagnosis but also during the evolution of the pathology, and to adjust any therapies “in itinere” [18,19,20]. 

This new role implies more extensive involvement of radiologists in multidisciplinary discussions and the planning of clinical-therapeutic pathways; it also implies a better understanding of molecular tests, resulting in the development of new diagnostic tools for better identification and localization of target lesions [21].

Tumor sampling through biopsy is essential for diagnostic and therapeutic decision making, and the appropriate biopsy technique depends on various factors, including tumor location and size [17,21].

Percutaneous biopsy guided using imaging modalities such as CT, CBCT, and PET/CT is a well-established and safe procedure with high accuracy rates [21]. Currently, the most widely used guidance imaging tool is fluoroscopy-CT (fluoro-CT), with an accuracy of more than 90% for malignant lung lesions, burdened by a low percentage of complications and minimal mortality [22]. In recent years, the use of Cone Beam Computed Tomography (CBCT), an imaging technique available on angiographic suites equipped with “C-arm”, has become widespread as guidance for percutaneous lung biopsies [21].

In small lesions, the goal remains to target the lesion and obtain the right amount of material for diagnosis; in larger lesions, it has been shown to be complicated to obtain samples with an adequate amount of material for diagnosis containing live cells. As demonstrated in the literature, the larger the lesion, the greater the number of repeated biopsies due to the presence of necrotic material [23]. 

Therefore, in large lesions, the use of a functional imaging guide remains mandatory to ensure a reduction in the number of inadequate biopsies and guarantee more vital tissue. 

Therefore, technology has allowed for the use of image fusion software in order to further improve the characteristics of individual imaging methods. 

Spectral CT could give an added value to the biopsy sampling (biomarkers information) and therefore to the clinician in the subsequent therapeutic choice. It has been our interest to allow the pathologist to formulate both a conventional histological diagnosis and the one we have defined as a “detailed histological diagnosis”, which consists of an in-depth study of immunohistochemical and molecular biology, to search for the predictive parameters of response to therapy in non-small cell lung cancer, because these examinations are not always feasible on standard biopsy samples due to the scarcity of vital material collected.

The aim of the study was to try to obtain more information on diagnostic samplings and biomarkers using dual-layer spectral CT in lung biopsies. Lung biopsies were performed by merging images obtained with CBCT with those from spectral CT to use them as functional guidance, experimenting with double sampling to determine the difference between the area with a higher Z-effective number and that with a lower Z-effective number. 

## 2. Materials and Methods

### 2.1. Patients Selection

This study was carried out at the Diagnostic and Interventional Radiology Department of Circolo Hospital, ASST Sette Laghi, Varese. 

All patients enrolled in the study provided informed consent, including for the publication of anonymous data. The procedures were conducted in compliance with the ethical standards set by the Institutional Research Committee and in accordance with the 1964 Declaration of Helsinki, including its subsequent amendments and ethical guidelines. Between March 2021 and July 2022, 10 consecutive patients were selected from a larger population of patients who underwent percutaneous transthoracic lung mass biopsy.

The thoracic oncology multidisciplinary board, which includes interventional radiologists, thoracic surgeons, oncologists, pulmonologists, nuclear medicine doctors, and pathologists, provided the clinical indication for biopsy.

The study included patients with the following criteria: age over 18 years, lung masses diameter larger than 3 cm, availability of a contrast-enhanced spectral CT scan performed within one month prior to the procedure, and signed informed consent allowing for the publication of anonymous data for scientific purposes.

**Figure 1:**
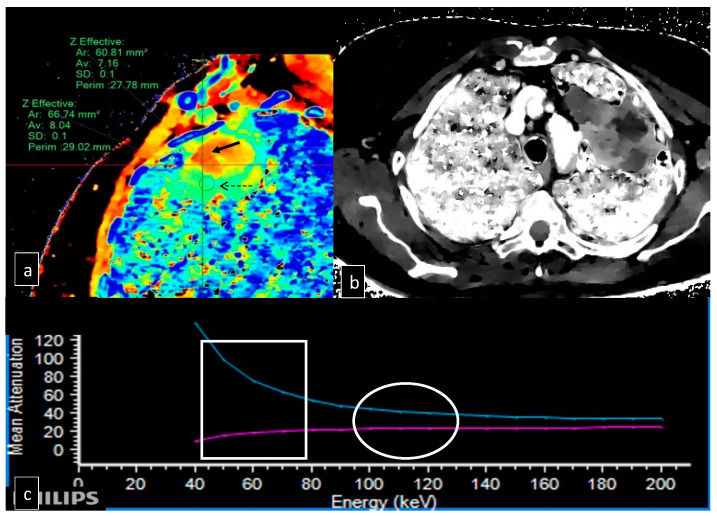
(**a**) Z effective sagittal reconstructions with placement of ROI (region of interest) in the most peripheral area of the lesion, with more cellular density (black dashed arrow) and one in the central part with high grade of fibrosis (black solid arrow), in this case, with 40 mL of iodinated contrast media, is well high lightened the differences between the two regions. (**b**) Gray-scale Z-effective axial reconstruction is technically mandatory for the fusion between the two systems but does not give any further information. (**c**) Histogram showing the correlation between mean attenuation (HU) and the energy (KeV) in the two areas with different Z-effective numbers; it is clear the two ROIs have high differences in the Z-effective map (square) but, at standard KeV (70–120), they have very similar behavior, making them nondistinguishable (circle).

The contrast-enhanced dual-layer spectral CT (iodinated contrast media: 40 mL; KeV: 70–120) was performed using our institutional scan protocol for thorax biopsy CT. The dimensions, localization in the lung lobe, and ROI with the maximum and minimum Z-effective number values were determined for each lesion. Z-effective spectral reconstructions were obtained (IQon, Philips Healthcare, Best, The Netherlands) (Figure 1).

The characteristics of the patients and the lesions (location, size, characteristics) were evaluated. We calculated all specifications related to lesion spectral data. All complications were evaluated and classified.

### 2.2. Technique of Execution of the Lung Biopsy

Lung biopsies were conducted using C-arm CBCT in combination with Virtual Navigation Software 12.1 (Allura Xper FD20, Philips, Amsterdam, The Netherlands) in an angiographic suite.

The specific techniques for the CBCT scan were previously presented [24]. The patient was positioned in the Angiosuite table considering the location of the lesion, accessibility, and distance from the cutaneous plane. A centering CBCT scan was performed initially to confirm and determine the precise location of the lesion.

Dual-layer spectral CT images were imported into the C-arm angiography workstation and overlaid on the centering CBCT using an automatic software and manual correction through anatomical reference points (XperCT Dual Release 3.5, Philips Healthcare, Amsterdam, The Netherlands). Subsequently, specific grey-scale Z-effective images were loaded to obtain a single fused Z-effective CBCT image.

**Figure 2:**
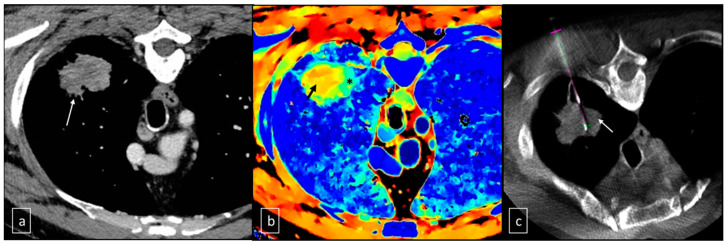
(**a**) Native axial CT acquisition, performed after intravenous administration of iodized contrast medium, in the venous phase demonstrates the hypodense pulmonary lesion (white arrow). (**b**) Z-effective axial reconstruction at the same level, which demonstrates reduced cellularity in the central portion of the lesion (color map from yellow to red—black arrow) and greater cellularity at the periphery (color map from green to blue—asterisk). (**c**) Intra-procedural axial CBCT image that demonstrates correct positioning of the cutting needle for sampling at the most peripheral portion of the lesion, probably with greater tumor cellularity, in the area of max Z value (white arrow). This sequence of images also shows the homogeneous pattern of the lesion tissue after contrast administration, so it was not discriminable the vital neoplastic tissue from the fibrotic one.

To correct the needle path, angiography navigation software (XperGuide Release 1.3, Philips Healthcare, Amsterdam, The Netherlands) was used. The fused Z-effective CBCT images facilitated the identification of regions with higher Z-effective content, as well as areas with poor cellularity (Figure 2). Real-time fluoroscopy overlapped with the needle path, enabling fluoroscopy/CBCT/Z-effective needle navigation.

For all procedures, a semi-automatic biopsy system with an 18-gauge coaxial trocar (Speedybell, Biopsybell, Modena, Italy) was used after skin disinfection and local anesthesia (7 ± 3 mL of 2% mepivacaine, Angelini, Rome, Italy).

**Figure 3:**
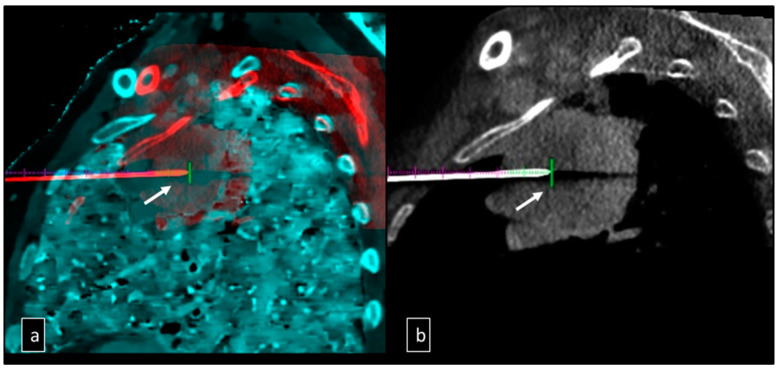
(**a**) Fusion of Z-effective images in gray-scale and CBCT images during a biopsy procedure of a large lesion located in the upper lobe with tissue differentiation; shows the tip of the needle in the deepest portion of the lesion in the low cellularity area for the first specimen (white arrow). (**b**) Sagittal CBCT shows the plain image during biopsy, with no difference inside the lesion, with the tip of the needle in the central portion of the lesion in the high vital tissue portion as demonstrated in the Z-effective reconstruction (white arrow).

Intraprocedural CBCT was conducted to verify the accurate positioning of the needle within the targeted lesion. As per practice, in large lesions, multiple samplings were performed. However, in this case, not randomly, but guided by spectral imaging indeed, almost two biopsy samples were obtained from high Z-effective (Z_max_) and low Z-effective (Z_min_) regions without requiring needle repositioning or new puncture at the skin level. Multiple cores were taken by retracting and moving the needle along its trajectory, using minimal tilting within the lesion (Figure 3). The needle and trocar were removed simultaneously, and a CBCT scan was conducted to detect any complications.

After the procedure, patients were monitored in the observation room by nursing staff for about 2 h. They then underwent antero-posterior chest X-rays to detect any distant post-procedural complications.

The collected material was sent to the Diagnostic Pathology Service for histological analysis. The Pathological Anatomy Unit analyzed the samples’ diagnostic potential using routine optical microscopy with dyes such as Alcian blue PAS and hematoxylin and eosin. Biomolecular characterization was also carried out, and the percentage of neoplastic cells, inflammatory cells, fibrosis, necrosis, or non-neoplastic lung parenchyma was evaluated. Additionally, the possibility of immunochemical and molecular investigations to determine the tumor’s biomolecular profile was assessed using the two different tissue samples.

### 2.3. Biopsy Analysis

**Figure 4:**
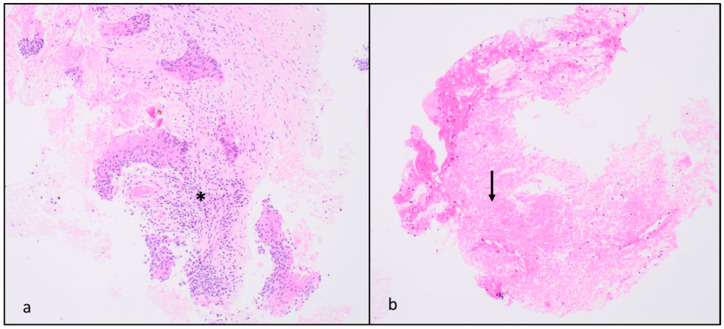
Anatomopathological slides (**a**) from the area with highest Z-effective number; this specimen shows sparse nests of squamous cell carcinoma in a dense fibro-hyaline stroma (about 70% of the material) [H&E ×10], asterisk. (**b**) The area with the lowest Z-effective number, shows extensive fibrinoid necrosis with a low number of tumor cells. [H&E ×10], black arrow.

The collected biopsy material was fixed in a solution of 10% buffered formalin at pH 7 for approximately 24 h. After fixation, the sample was embedded in paraffin and sectioned into 3 μm thick slices. Hematoxylin and eosin as well as Alcian blue PAS stains were then performed for routine diagnosis. When characterizing the disease’s prognostic factors for therapeutic purposes was necessary, the suitability of the material was evaluated (ensuring a well-preserved sample with at least 100 cells of neoplastic cellularity) and images were taken accordingly (Figure 4). 

For immunohistochemical study of PDL-1 and ALK receptors, additional 3 μm sections were prepared and processed using the mouse monoclonal anti-PDL-1 (clone 22C3, dilution 1:50, Dako, Glostrup, Denmark) and the monoclonal antibody in rabbit anti-ALK (clone D5F3, Roche Diagnostics, Ventana, Tucson, Arizona) on the BenchMark Ultra immunostainer (Roche Diagnostics, Ventana).

For the molecular study of EGFR, tumor tissue was obtained from 3 histological sections of 8 μm using manual microdissection to ensure a minimum of 100 tumor cells and a percentage of neoplastic nuclei greater than 10%. Tumor DNA was extracted using the Maxwell^®^ Automated Extractor and the Maxwell^®^ 16 FFPE Plus LEV DNA Purification Kit (Promega, Corporation, Madison, WI, USA). EGFR gene mutation analysis was carried out using the Miriapod Lung Status (Diatech Pharmacogenetics, Jesi, Italy) kit and MALDI-TOF (Matrix Assisted Laser Desorption Ionization Time-of-Flight) mass spectrometry. Finally, the sections underwent FISH analysis for the cytogenetic study of the ROS gene using break-apart-type probes (Abbott Molecular, Abbott Park, IL, USA and Dako, Glostrup, Denmark). Each case was independently studied by two operators following a detailed scoring system.

### 2.4. Data Analysis and Statistics 

Technical success was defined as the complete execution of the lung biopsy procedure, with subsequent histopathological confirmation of the lesion with a correct diagnosis. The possibility of performing a correct immunohistochemical and molecular characterization of the tumor was also evaluated on the samples taken from the region with the lowest Z-effective number and the highest Z-effective number.

The technical success and feasibility of conducting immunohistochemical and molecular biology investigations were evaluated using a 2 × 2 contingency table and Fisher’s test. The agreement between the biopsy results and the histological analysis of the surgical specimen was assessed using Spearman’s Rho correlation coefficient.

SPSS version 25.0 (IBM, Armonk, New York, NY, USA) was used for all statistical analyses. In all cases, two-tailed tests were used. *p*-values were considered significant when <0.05. Data collection was performed using an informatic spreadsheet of anonymous data (Excel 16.74, Microsoft, Redmond, WA, USA).

## 3. Results

Of the ten patients included in the study, six were male and four were female. The mean age of the population was 65 years (range: 34–85). The lesions were located in the left upper lobe (six), right lower lobe (two), right upper lobe (one), and middle lobe (one). The mean diameter of the biopsied lesions was 77 mm (range: 31–154). The maximum Z-effective number value was 8.10 (range 7.92–8.30) at the sampling points where the ROI for the evaluation of the Z-effective value was positioned. The minimum Z-effective number value was 7.41 (range 7.13–7.82) (Table 1).

The most common post-procedural complication was pneumothorax, which occurred in three out of ten patients at the post-procedure X-ray control. No peri-lesional bleeding suffusions were noted, and pleural drainage was not required in any case to treat the pneumothorax.

All lesions were subjected to histopathological evaluation, with the detection of malignancy in 10 out of 10 samples (4 squamous cell carcinomas, 3 adenocarcinomas, 2 lymphomas, and 1 sarcoma) in the samples taken from the Z*_max_* areas; in the Z*_min_* areas, malignant tumor cells were found in 7 out of 10 samples (3 squamous cell carcinomas, 3 adenocarcinomas, and 1 lymphoma), while 3 procedures were non-diagnostic (Table 2).

Technical success was therefore achieved in 100% of cases for Z*_max_* sampling and in 70% for Z*_min_* sampling (*p*-value: 0.2105 by Fisher’s exact test). In three cases, the material collected in the area with the lowest Z-effective number was found to be too poor in cellularity to enable the correct histological diagnosis.

The diagnostic capacity to detect immunohistochemical biomarkers was also evaluated: in the samples taken from the Z*_max_* areas, it was possible to characterize the lesions from an immuno-molecular point of view in 9/10 (90%) cases, while in those with low Z-effective numbers, 4/10 (40%) cases (*p*-value: 0.0573 by Fisher’s exact test).

The quantity of neoplastic, inflammatory, fibrotic, and necrotic material and healthy lung parenchyma was identified from the various samples, expressed as percentages in Table 2.

In the case of biopsy samples taken from the Z*_max_* area of the lesion, higher percentages of neoplastic cells were found compared to the samples taken from the Z*_min_* area of the lesion in 8 cases out of 10, specifically, 80% versus 40%, 90% versus nondiagnostic sample, 25% versus 10%, 40% versus nondiagnostic sample, 80% versus 30%, 80% versus 5%, 90% versus nondiagnostic sample, and 40% versus 25%. In 1 out of 10 cases, the tumor cellularity found in the 2 samples was equivalent (60%, in 1 case of adenocarcinoma). 

On the other hand, the findings of tumor fibrosis and necrosis were detected in slightly higher percentages in the samples obtained from the lesion area with the lowest Z-effective number. 

Furthermore, in the case of the biopsy sample taken from the Z*_max_* zone, it was possible for the pathologist to perform a correct biomolecular and immunohistochemical typing in 9 out of 10 cases, while in the biopsy sample taken from the Z*_min_* area, this characterization was possible only in 4 out of 10 cases (Table 3) [25,26].

## 4. Discussion 

Thoraco-pulmonary lesions are characterized by poor clinical representation and, as a result, can still be diagnosed at an advanced stage and therefore identified when they are already large masses. 

Like all neoplastic lesions, even if their appearance is clearly attributable to a tumor, they require a biopsy for tissue typing to perform a biomarker profile and apply custom therapies [23]. 

In the management of lung nodules, current guidelines emphasize the size of the lesion as the primary criterion for biopsy. Studies have shown that the probability of malignancy increases proportionally with the size of the lesion [27].

Therefore, for nodules smaller than 8 mm, which have a low probability of malignancy and pose challenges for core needle biopsy, imaging surveillance using CT and/or PET is recommended. The frequency of follow-up evaluations should be determined based on additional factors such as stability over time, individual characteristics, and clinical judgment, as reported by Gould et al. [28]. 

Kothary et al. have found that to obtain an accurate biopsy with reliable cytopathological results, the minimum diameter suggested is 1.5 cm [29,30]. Li et al. also demonstrated that the diagnostic accuracy of CT-guided percutaneous needle aspiration biopsy for large lesions (>1.5 cm) was 96%. In comparison, the diagnostic accuracy for small nodules (<1.5 cm) was 74%, and this difference was found to be statistically significant with a *p*-value of less than 0.05 [30].

Lesions above 5 cm have a dual nature: on the one hand, they are technically easy because their large size makes the tumor easily puncturable, but, on the other hand, they are often characterized by a cellular and tissue structure that is heterogeneous with fibrotic and necrotic parts, and perhaps only a small percentage of the lesion volume consisting of vital neoplastic cells. 

This small portion is what guarantees the success of the anatomopathological analysis as it is the only one with active cells that express receptors and present a profile on which an effective therapy can be based [23]. 

Functional and metabolic imaging techniques such as spectral CT or PET provide valuable insights during biopsy procedures by identifying regions with a higher concentration of neoplastic cells. 

Fontana et al.’s attempt to use functional imaging as a guide for biopsies dates back a long time; initially, they experimented with the use of PET-CT through fusion with CBCT to obtain a functional guide in lung lesions without distinction of size, and they saw how it worked in lesions larger than 3 cm. 

They published a paper where two groups of patients were subjected to two different guiding methods for biopsy procedures (CBCT and fusion of PET/CT with CBCT) [31]. 

All the results showed that percutaneous biopsies with fused PET/CT-CBCT images allow for the reduction of the number of inadequate samples but, in small lesions, there was poor utility from either a technical or tissue analysis point of view. The PET/CT-CBCT-guided biopsy group demonstrated higher rates of both technical and clinical success compared to the CBCT-alone-guided group (98.9% vs. 93.3% and 98.9% vs. 95.2%, respectively). Subsequently, the same group published another paper about double sampling in large lesions using PET-CT as functional imaging guidance [32]. In these specific cases, the functional guide gave excellent results. The technical success rate was 100% for the samples obtained from SUV_max_, and 70% for the samples obtained from SUV_min_. Upon pathological evaluation, the first group (SUV_max_ samples) showed higher percentages of neoplastic cells. The biomolecular profile was successfully obtained in 100% of cases in the first group and a correlation between the standardized uptake value and the technical success of the biopsy sample has been identified.

PET-CT has limitations regarding blood glucose levels, waiting times, and machine availability, making it less effective in today’s fast-paced medicine. To overcome these limitations, our center has considered using spectral computed tomography (spectral CT) as a potential replacement for PET-CT. Spectral CT is an advanced technology that utilizes a dual-energy detector (DECT) with two layers to collect low and high-energy data simultaneously during standard CT protocols [33]. The dual-layer setup consists of upper and lower layers made of garnet scintillator yttrium and gadolinium oxysulfide, respectively. Each layer’s detectors convert light into electrical signals, which are then digitally processed. The gantry rotation time is 270 ms [34,35]. Spectral CT allows for various reconstructions, including “Z-effective” images based on the effective atomic number [36]. These color-coded images differentiate different components of a lesion based on their atomic number, providing higher discrimination than attenuation in Hounsfield units (HU) [37,38]. The Z-effective images compress data into a dynamic range, visually represented in an RGB color scale. Z-effective images, iodine maps, and “uric acid pair” images are utilized for various evaluations, such as urinary and biliary stones, gout, lesion and plaque characterization, tumor tissue or organ perfusion, tumor response to therapy, and virtual colonoscopy without adequate bowel preparation [33,39]. Dual-layer spectral CT offers advantages over conventional dual-energy technology, including the availability of spectral data to all patients without workflow changes. It also allows for the application of radiation dose reduction strategies and has no restrictions on the visual field or gantry rotation time. However, a drawback of spectral technology is the requirement of a tube potential of at least 120 kVp for sufficient spectral separation. This may limit radiation dose reduction for smaller pediatric patients, and contrast resolution is reduced in conventional images due to high kVp values [40].

Spectral imaging offers advantages such as the ability to fuse images and the minimal amount of contrast required to differentiate tissues.

Therefore, incorporating spectral/CT-CBCT fusion imaging in this context would not only improve the effectiveness of biopsy sampling but also reduce the need for multiple procedures with inconclusive outcomes.

Before starting this study, Fontana et al. conducted a thorough evaluation of various reconstruction techniques and imaging combinations. This investigation led to the conclusion that the most accurate results were obtained through the use of combined Zeff map/CBCT fusion imaging [41].

The results obtained from the fusion of the Z-map (in grayscale; necessary to make the series readable for the C-arm) were excellent both in terms of visual identification of the lesion and in terms of tissue structure analysis, as demonstrated by the graphs presented in the paper. Indeed, the area with a high Zeff corresponds to the concentrated presence of cells or structures with greater atomic numbers, thus to a tissue region with a higher percentage of viable neoplastic cells; in contrast, regions with lower Zeff imply the likelihood of less densely populated cellular structures or elements with lower atomic numbers. These regions might correspond to locations characterized by greater instances of necrosis, inflammation, and fibrosis, thus to a lower presence of neoplastic cells.

The peculiarity lies that the Z-map, as already demonstrated in the literature, provides information regarding not only the atomic number but also the density in absolute terms of the tissue density and that this, used as a functional guide in biopsies, guarantees a better result in tissue typing.

Several studies have examined the use of spectral CT to improve lesion identification in various organs but not as biopsy guidance. For example, Kim et al. demonstrated how reconstructions based on iodine concentration can serve as an alternative to PET-FDG in the study of lymphomas [42]. In this study, there is a notable association between metabolic activity, evaluated through FDG-PET/CT, and iodine concentration, assessed through dual-layer spectral CT (SDCT), in malignant lymphomas. Additionally, there is a significant correlation between iodine concentration and SUV_max_ in lymphoma patients, further supporting the potential of iodine concentration as an imaging biomarker for detecting lymphoma activity.

Other authors, always as an imaging-improving tool but not an interventional tool, have evaluated the possibility of obtaining improved detection of prevertebral hematoma using electron density, suggesting that spectral CT may enhance hematoma detection [43]. 

This study included 38 patients with post-traumatic cervical spine imaging. Both SDCT and MRI examinations were performed, with MRI used as the reference standard. The study compared combined conventional/electron density (C + ED) images to conventional CT (CCT) images alone. A total of 18 prevertebral hematomas were identified.

The sensitivity of CCT ranged from 33% to 50%, with a specificity of 75% to 80%. In comparison, the sensitivity of C + ED reconstructed images was 77% to 83%, with a specificity of 85% to 90%. The overall accuracy increased from 55% to 66% with CCT alone, to 84% when using C + ED images.

The study concluded that the use of combined conventional and electron density reconstructions (C + ED) in SDCT improved the diagnostic accuracy for detecting post-traumatic prevertebral hematoma compared to conventional images alone.

Another study published by Zheng et al. in the *European Journal of Radiology* in 2018 investigated the utility of spectral CT to differentiate malignant and benign pulmonary nodules. 

This study involved 60 patients with pulmonary nodules, including 39 malignant and 21 benign nodules. Spectral imaging chest contrast CT scans were performed during the pulmonary arterial phase (PP), arterial phase (AP), and venous phase (VP). The iodine concentrations of proximal and distal regions within the nodules were recorded using iodine-based material decomposition images. 

The researchers found that spectral CT imaging, in this case, iodine-based and water-based material decomposition images, provided improved lesion conspicuity and better visualization of small vessels [44]. Therefore, in the literature, we can find few studies describing the importance of spectral CT in various fields and anatomical regions, including the study of brain gliomas [45], liver fibrosis [46], urological [47] and gynecological tumors [48], spinal tophaceous gout [49,50], and many others [33,34,51]. 

On the other hand, being a relatively new cutting-edge technique, its use as a guide for biopsies has been poorly investigated.

When it comes to biopsy procedures, accurate localization and targeting of the lesion are crucial for achieving diagnostic precision and minimizing complications. Spectral CT, as previously mentioned, can enhance the biopsy process by providing additional information, even compared with PET/CT, about tissue composition and vascularity, aiding in lesion characterization and improving targeting accuracy.

However, in the literature, there are very few studies specifically addressing this aspect. Yamamoto et al. describe a case report on electron density (ED) maps for the detection of bone lesions, specifically in cases of breast carcinoma, on which subsequent biopsies were performed using spectral imaging [25]. A 61-year-old woman with a history of breast cancer surgery underwent a spectral CT-guided bone biopsy of the right ilium using ED maps, which resulted in the diagnosis of breast cancer metastases in the intertrabecular bone. A comparison between the ED maps and the pathological specimen revealed that areas with high ED values were exclusively found within the tumor area, indicating high cellularity. This study highlights the potential usefulness of ED maps generated by DECT in accurately identifying bone metastases with high cellularity, especially in intertrabecular bone metastases, which are occasionally referred to as CT-negative bone metastases.

Another study recruited patients with suspected lung cancer: 41 patients were enrolled to identify the region of high tumor cell percentage, and then an additional 15 patients were recruited to validate the accuracy of the high tumor cell proportion region (HTPR). In each of the 41 patients, suspicious regions with high or low tumor cell proportions were targeted for separate biopsies based on local iodine density (IoD) values [26]. 

This study aimed to evaluate the use of spectral CT parameters in identifying the HTPR in lung cancer tumors prior to transthoracic lung biopsy (TTLB). Significant correlations were found between tumor cell proportions and spectral CT parameters. The median tumor cell proportions obtained in the HTPR, and low tumor cell proportion region (LTPR) were 57.5% and 12.5%, respectively, indicating the feasibility of using spectral CT parameter values to plan the puncture path for TTLB.

In distinguishing intratumor HTPR with a tumor cell proportion using an IoD value of ≥0.59 mg/mL as a single quantitative criterion, 97% of the specimens had a tumor cell proportion of ≥20%. These findings were also confirmed in a validation test with a small sample size, where 100% of the specimens had a high tumor cell proportion (≥20%).

The results suggest that spectral CT IoD can be utilized to identify regions with a high tumor cell proportion for biopsy purposes. They concluded that spectral CT parameters can be used to identify regions with at least 20% tumor cell content in lung cancer for biopsies [26].

Spectral CT, with its ability to provide enhanced tissue characterization and differentiation, holds significant promise as a guiding tool for biopsies in the future. This technology has the potential to revolutionize the way biopsies are conducted, improving accuracy, diagnostic yield, and patient outcomes.

In the near future, spectral CT could improve interventional procedures (as spectral CT evolves, the potential to seamlessly integrate biopsy guidance with therapeutic interventions grows; clinicians could simultaneously visualize, target, and treat lesions during the same procedure, optimizing patient care and outcomes) or even be combined with artificial intelligence (the integration of AI algorithms with spectral CT could further refine biopsy guidance; AI can assist in real-time image analysis, highlighting regions of interest, predicting tissue composition, and ultimately aiding physicians in making informed decisions).

The future objective remains to find a spectral “tool” that allows us to make this distinction even without the use of contrast, which, for now, remains the Achilles’ heel of this procedure.

The study has several limitations, including a small number of patients, all with lung parenchymal lesions; therefore, there were no findings on tumors in other areas; there is no direct comparison with PET/CT regarding the subjective evaluation of cells with Z-effective; all procedures were performed by only two operators; there is no existing standard in the literature to reference; and there is no standardization of Z-effective values in the literature in relation to tissue density/cellularity.

## 5. Conclusions

Furthermore, despite the over-expressed limitations and the need for further data based on a larger cohort of patients, our findings on the role of dual-layer spectral CT and the usefulness of color-coded Z-effective images are very encouraging, particularly regarding biomarkers information in the era of patient-tailored oncologic therapy. 

## Figures and Tables

**Table 1:** Characteristics of patients and lesions subjected to percutaneous biopsy (antero-posterior, cranio-caudal, and latero-lateral diameters expressed in centimeters, localization, minimum, and maximum Z-effective number value).

Patients	Gender	Age	AP Diameter (cm)	CC Diameter (cm)	LL Diameter (cm)	Location	Z Effect max	Z Effect min
**1**	F	58	4.1	8.0	3.5	RLL	8.21	7.59
**2**	M	76	11.5	10.0	9.1	RLL	8.10	7.30
**3**	M	34	10.2	15.4	13.0	LUL	7.92	7.50
**4**	F	62	8.4	9.3	9.9	RML	8.02	7.15
**5**	M	79	8.1	9.4	7.9	LUL	8.23	7.40
**6**	F	71	3.1	3.4	3.3	LUL	8.30	7.79
**7**	M	80	9.8	4.9	3.7	LUL	8.29	7.82
**8**	M	42	10.2	16.5	11.5	LUL	8.29	7.15
**9**	F	64	5.1	4.9	6.2	LUL	7.98	7.13
**10**	M	85	8.2	11.6	9.0	RUP	8.10	7.38

**Table 2:** Results in the percentage (%) of biopsied tissue of lung lesion performed in the lesional parenchyma area with higher and lower Z-effective numbers.

	High Z Effective					Low Z Effective				
Patient	Neoplasia	Flogosis	Fibrosis	Necrosis	Lung Parenchyma	Neoplasia	Flogosis	Fibrosis	Necrosis	Lung Parenchyma
**1**	80	10	0	10	0	40	0	30	30	0
**2**	90	5	0	5	0	non diagnostic				
**3**	25	60	15	0	0	10	80	10	0	0
**4**	40	5	10	45	0	non diagnostic		80	20	
**5**	10	5	70	10	5	20	0	0	80	0
**6**	80	5	15	0	0	30	5	65	0	0
**7**	80	5	15	0	0	5	15	75	0	5
**8**	90	0	5	5	0	non diagnostic				
**9**	60	25	0	15	0	60	10	0	30	0
**10**	40	5	50	5	0	25	5	70	0	0
**Mean**	59.5	12.5	18	9.5	0.5	30.83	16.43	35.71	20	0.71
**SD**	28.91	17.99	23.48	13.43	1.58	17.44	28.54	33.72	30	1.89
**Spearman’ Rho**	0.25944	0	0.10385	0.8454	−0.16667					
*p-value*	0.57424	1	0.82465	0.01658	0.72097					

SD: Standard Deviation. Note that a >0.05 *p*-value stands for a statistically significant difference (absence of correlation) among the investigated groups.

**Table 3:** Histopathological diagnosis of lung biopsies and execution of immunomolecular analysis for the samples obtained in the tumor region with the highest and lowest Z-effective number.

Patients	Diagnosis	High Z Effect	Low Z Effect
		*Biomolecular Characterization*	*Biomolecular Characterization*
**1**	Squamous cell carcinoma	yes	yes
**2**	Sarcoma (single sampling)	yes	NO
**3**	Hodgkin lymphoma	yes	NO
**4**	Squamous cell carcinoma	NO	NO
**5**	Squamous cell carcinoma	yes	NO
**6**	Squamous cell carcinoma	yes	yes
**7**	Adenocarcinoma	yes	yes
**8**	Diffuse large B-cell lymphoma (single sampling)	yes	NO
**9**	Adenocarcinoma	yes	NO
**10**	Adenocarcinoma	yes	yes

## Data Availability

Data is contained within the article.
